# Effects of bed rest and immobilization on intramuscular and intermuscular adipose tissue: A systematic review

**DOI:** 10.1113/EP093306

**Published:** 2026-02-23

**Authors:** Konstantinos Prokopidis, Julia Margarita Reyes, Gustavo Duque

**Affiliations:** ^1^ Department of Musculoskeletal and Ageing Science, Institute of Life Course and Medical Sciences University of Liverpool Liverpool UK; ^2^ Bone, Muscle & Geroscience Group, Research Institute of the McGill University Health Centre McGill University Montreal Quebec Canada; ^3^ Geriatrics Section Clinical Hospital University of Chile Santiago Chile; ^4^ Department of Medicine McGill University Montreal Quebec Canada

**Keywords:** bed rest, immobilization, intermuscular fat, intramuscular fat, muscle disuse

## Abstract

Muscle disuse caused by bed rest and immobilization is associated with muscle atrophy and insulin resistance, which might be related to increases in intramuscular fat (IntraMAT) and intermuscular fat (InterMAT) accumulation. In this systematic review, we compiled evidence on the effects of bed rest and unilateral lower‐limb immobilization on IntraMAT and InterMAT in healthy adults. Following PRISMA guidelines, we searched PubMed, Scopus, Web of Science, and Cochrane Library from inception until July 2025. Included studies were clinical trials involving healthy adults (≥18 years old) undergoing bed rest or immobilization, with IntraMAT and InterMAT as outcomes (CRD420251112427). Two reviewers extracted data and assessed bias using RoB2 and ROBINS‐I tools for randomized and non‐randomized studies, respectively. Data were synthesized using a narrative synthesis. Nine studies met the inclusion criteria. Bed rest increased IntraMAT in the lumbar multifidus, erector spinae, and quadratus lumborum by 18.7%, 5.4% and 4.5%, respectively (*P* < 0.01), whereas thigh‐related changes were inconsistent. Immobilization produced heterogeneous effects: calf muscles showed both small decreases (−0.5%) and increases (+0.3%), whereas thigh IntraMAT increased by 12.8% after 28 days. InterMAT increased in the lumbar spine (1.2–5.3%) and immobilized thigh (+14.5%) and calf (+20%) muscles. Muscle disuse from bed rest or immobilization can promote site‐specific increases in IntraMAT and InterMAT, particularly in lumbar regions, highlighting muscle fat infiltration as a potential key adaptation to unloading.

## Abstract (highlights)

- **What is the topic of this review?**
  What is the impact of bed rest or immobilization on site‐specific changes in intramuscular (IntraMAT) and intermuscular (InterMAT) fat in healthy adults?
- **What advances does it highlight?**
  Bed rest may increase IntraMAT in lumbar muscles (by up to 18.7%), with variable responses in the thigh, and immobilization could significantly increase thigh IntraMAT (by 12.8%), indicating site‐specific fat infiltration patterns that depend on the disuse model. InterMAT accumulation might be more pronounced in the lumbar spine (1.2–5.3%) during bed rest and in the thigh (+14.5%) and calf (+20%) during immobilization, highlighting muscle disuse as a driver of muscle fat deposition, with implications for muscle atrophy and insulin resistance.

## 1 INTRODUCTION

Physical inactivity, particularly various models of muscle disuse, such as prolonged bed rest or immobilization, can induce rapid and significant physiological losses of skeletal muscle tissue. Several mechanisms might mediate muscle disuse‐induced atrophy, underpinning reduced signalling of muscle protein synthetic responses and altered expression of mitochondrial and neuromuscular adaptations (Deane et al., 2024).

For instance, a 5‐day period of bed rest can be sufficient to cause muscle strength and mass loss, in addition to increased fall risk, further indicating potential disruption of muscle fibre mechanical properties (Marusic et al., 2021). These effects can be especially important in adults undergoing critical care or who are hospitalized, given that the physiological impact of secondary conditions or catabolic states (such as surgery) may worsen muscle health (Lees et al., 2025; Parry & Puthucheary, 2015). Among these adaptations, changes in intramuscular fat (IntraMAT; consisting of lipid droplets within muscle fibres) and intermuscular fat (InterMAT; located between muscle fibres or fascicles) have garnered increasing attention owing to their implications for cardiometabolic and muscle metabolism (Wang et al., 2024). Although excessive accumulation of these adipose tissues during periods of inactivity could contribute to insulin resistance and reduced muscle mass and strength (Di Girolamo et al., 2021; Eggelbusch et al., 2024), evidence on whether their accumulation alone is responsible for these effects requires further investigation (Dirks et al., 2016).

The muscle disuse‐induced build‐up of IntraMAT and InterMAT is believed to result from an imbalance between lipid uptake, storage and oxidation, in addition to altered adipocyte differentiation, driven by reduced mitochondrial activity and impaired insulin signalling (Addison et al., 2014; Eggelbusch et al., 2024). These changes may have functional consequences, including compromised muscle contractility, decreased oxidative capacity, and increased systemic inflammation (Carter et al., 2019). Currently, the impact of bed rest and immobilization on IntraMAT and InterMAT, along with their effects on various fat‐infiltrated muscle tissues, has not been examined systematically, despite their physiological significance. Understanding site‐specific fat changes is crucial, because IntraMAT and InterMAT differentially affect muscle quality and metabolic health. This systematic review aims to synthesize existing evidence on the effects of bed rest and immobilization on both IntraMAT and InterMAT in different skeletal muscle tissues of healthy adults.

## 2 MATERIALS AND METHODS

This systematic review was conducted in accordance with the Preferred Reporting Items for Systematic Reviews and Meta‐Analyses (PRISMA) guidelines (Page et al., 2021). The protocol was registered in the International Prospective Register of Systematic Reviews (PROSPERO; CRD420251112427).

### 2.1 Search strategy

Two independent reviewers (K.P. and J.M.R.) searched PubMed, Scopus, Web of Science and the Cochrane Library from inception until July 2025. The comprehensive search strategy used is described in Table 1. A manual search of references cited in the selected articles and published reviews was also performed. Discrepancies in the literature search process were resolved by a third investigator (G.D.). Studies were included based on the following criteria: (1) must be a clinical trial, either randomized or non‐randomized; (2) included participants with a mean age of ≥18 years; (3) participants were overall healthy, without reporting any major comorbidities; and (4) participants were not receiving any type of intervention. Studies were excluded if they: (1) were non‐clinical trials (i.e., observational studies); (2) included participants with major comorbidities or participants who were hospitalized or critically ill; or (3) a full text was not available and not in English.

**TABLE 1:** Study and participant characteristics of the included studies.

Database	Search terms
PubMed	(fat infiltration OR intramuscular fat OR intramuscular adipos* OR muscle fat OR intermuscular fat OR intermuscular adipos* OR “IMAT” OR myosteatosis OR ectopic fat OR lipid infiltration) AND (immobili* OR bed rest OR bedridden OR head down tilt OR limb suspension OR muscle disuse)
Cochrane Library	(fat infiltration OR intramuscular fat OR intramuscular adipos* OR muscle fat OR intermuscular fat OR intermuscular adipos* OR “IMAT” OR myosteatosis OR ectopic fat OR lipid infiltration) AND (immobili* OR bed rest OR bedridden OR head down tilt OR limb suspension OR muscle disuse)
Web of Science	(fat infiltration OR intramuscular fat OR intramuscular adipos* OR muscle fat OR intermuscular fat OR intermuscular adipos* OR “IMAT” OR myosteatosis OR ectopic fat OR lipid infiltration) AND (immobili* OR bed rest OR bedridden OR head down tilt OR limb suspension OR muscle disuse)
Scopus	(intramuscular fat OR intramuscular adipose tissue OR muscle fat OR intermuscular fat OR intermuscular adipose tissue OR lipid infiltration OR myosteatosis OR ectopic fat) AND (bedridden OR head down tilt OR bed rest OR immobilization OR immobilisation)

### 2.2 PICOS criteria

Population: Healthy adults ≥18 years of age.

Intervention: Bed rest or unilateral lower‐limb immobilization for a minimum of 7 days.

Comparator: Baseline changes or changes observed in comparison to the non‐immobilized lower limb.

Outcomes: InterMAT and IntraMAT of various skeletal muscle tissues. These could include the lumbar spine, the thighs, quadriceps and calves, and/or the psoas muscle.

Study design: Randomized and non‐randomized trials.

### 2.3 Data extraction

Two authors (K.P. and J.M.R.) extracted data regarding the date of publication, study design, participant health status and sample size, age, body mass index, body weight, sex, tool of InterMAT and IntraMAT assessment, model of muscle disuse alongside its duration, and outcomes of interest (i.e. area of muscle fat infiltration).

### 2.4 Risk‐of‐bias assessment

The quality of included studies was assessed using the Cochrane Risk‐of‐Bias 2 (RoB2) and the Risk Of Bias In Non‐randomized Studies—of Interventions (ROBINS‐I) tools for randomized controlled trials (Higgins et al., 2011) and non‐randomized controlled trials (Sterne et al., 2016), respectively, and were evaluated by two independent reviewers (K.P. and J.M.R.). Appraisal of bias risk using the RoB2 tool included assessment of the following domains of bias in randomized clinical trials: randomization process; deviations from intended interventions; missing outcome data; measurement of the outcome; and selection of the reported result. If studies had a crossover design, a RoB2 modified tool was used, with the additional domain related to bias arising from the timing of identification and recruitment of individual participants in relationship to the timing of randomization. According to the scoring system of the tools, study quality was defined as low, some concerns or high risk of bias.

### 2.5 Data synthesis

Quantitative data were treated as continuous measurements, and any standard errors (SE) were transformed to standard deviations (SD), using the formula: SE = SD/√*n* or SE = (upper 95% confidence interval (CI) limit − lower 95% CI limit/2 × 1.96) to enhance uniformity across studies, facilitating the interpretation of the systematic review through a narrative synthesis.

## 3 RESULTS

**FIGURE 1:**
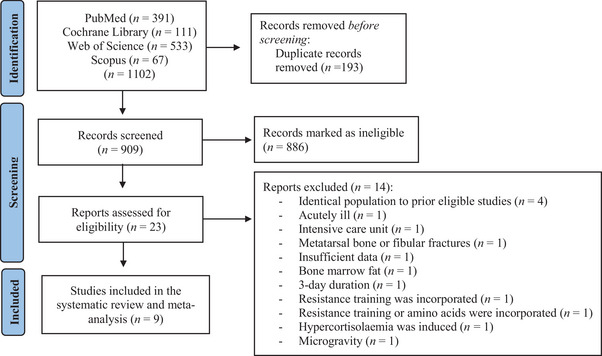
Flowchart of the study screening and eligibility.

The initial literature search displayed 1102 publications. Following the exclusion of 193 duplicates, 909 unique publications were screened, from which 886 were marked as ineligible after reading their titles and abstracts. In total, 23 full texts were screened, and nine of them were deemed eligible for inclusion in this systematic review and meta‐analysis (Bergouignan et al., 2009; De Martino et al., 2021; Fuchs et al., 2024; Gerlach et al., 2017; Holloway et al., 2019; Manini et al., 2007; Ogawa, Belavý et al., 2020; Rudwill et al., 2018; Wesselink et al., 2025; Figure 1). From the 14 studies that were excluded, four studies included identical populations to prior eligible studies (De Martino et al., 2022a, 2022b; McNamara et al., 2019; Tran et al., 2021), one study included acutely ill patients (Rommersbach et al., 2020), one study included patients admitted into an intensive care unit (Erley et al., 2024), one study included participants with metatarsal bone or fibular fractures (Yoshiko et al., 2018), one study had insufficient information (Yuri et al., 2021), one study had a duration of 3 days (Pagano et al., 2018), one study measured bone marrow fat (Trudel et al., 2019), in one study resistance training was incorporated (Standley et al., 2017) and in another study resistance training or amino acids were incorporated in the study arms (Brooks et al., 2008), in one study hypercortisolaemia was induced (Cree et al., 2005), and one study used microgravity (Demangel et al., 2017). Characteristics of the included studies are shown in Table 2.

**TABLE 2:** Search terms employed in the screening based on title, abstract, and keywords in the literature search.

First author, year	Outcome	Intervention (male/female)	Age (years), range or (SD)	BMI (kg/m^2^)	Bod weight changes (kg)	Protocol	Duration (days)	Assessment tool	Dietary intake control	Fat infiltration (pre)	Fat infiltration (post)	Change
Bergouignan et al., 2009	Calf IntraMAT, %	0/8	34 (4)	21.3 (1.4)	−3.3	Bed rest	60	MRI	Yes	–	–	Gastrosoleus: +2.7% (*P* = 0.54)
De Martino et al., 2021	Lumbar InterMAT, %	6//2	32–34	–	−1.8 (1.3)	Bed rest	59	MRI	Yes	Lumbar multifidus: (L5/S1): 24.1 (8.93) (L4–L5): 20.7 (10.6) (L3–L4): 15.2 (6.5) (L2–L3): 12.6 (6.9) (L1–L2): 14.0 (7.4) Lumbar erector spinae: (L1–L2): 9.4 (3.4) (L2–L3): 11.3 (4.8) (L3–L4): 12.7 (6.6) (L4–L5): 21.2 (12.6) (L5–S1): 37.8 (13.3) Quadratus lumborum: (L1–L2): 82. (2.4) (L2–L3): 8.7 (2.7) (L3–L4): 8.1 (1.9) Psoas major: (L1–L2): 10.2 (2.1) (L2–L3): 10.3 (1.6) (L3–L4): 10.3 (2.2) (L4–L5): 11.5 (1.6) (L5–S1): 11.1 (1.7)	Lumbar multifidus: (L5/S1): 29.4 (9.2) (L4–L5): 23.5 (10.9) (L3–L4): 17.4 (6.8) (L2–L3): 13.8 (7.1) (L1–L2): 15.2 (8.8) Lumbar erector spinae: (L1–L2): 11.1 (4.3) (L2–L3): 13.7 (6.2) (L3–L4): 15.5 (8.0) (L4–L5): 24.3 (12.8) (L5–S1): 41.1 (15.1) Quadratus lumborum: (L1–L2): 8.3 (2.4) (L2–L3): 8.6 (2.0) (L3–L4): 7.6 (1.9) Psoas major: (L1–L2): 9.2 (1.6) (L2–L3): 9.5 (1.6) (L3–L4): 10.4 (1.9) (L4–L5): 11.5 (1.9) (L5–S1): 10.7 (1.4)	
Fuchs et al., 2024	Anterior thigh IntraMAT (quadriceps femoris, sartorius and tensor fascia latae), %	12/0	24 (3)	23.7 (3.1)	−2.1 (1.1)	Bed rest	14	MRI	Yes	4.4 (1.2)	4.5 (1.4)	*P* = 0.023
Gerlach et al., 2017	Calf IntraMAT, %	5/0	27.3 (3.9)	23.0 (1.5)	–	Unilateral limb suspension	60	MRI	–	Gastrocnemius medialis: 10.2 (1.2) Gastrocnemius lateralis: 10.8 (1.4) Soleus: 10.9 (1.5)	Gastrocnemius medialis: 9.9 (1.2); *P* > 0.05 Gastrocnemius lateralis: 10.3 (1.0); *P* < 0.05 Soleus: 11.2 (2.0); *P* > 0.05	
Holloway et al., 2019	Thigh IntraMAT, %	10/0	–	–	−0.2	Unilateral limb suspension	28	MRI	Yes	–	–	+12.8 (6.1); *P* < 0.01
Manini et al., 2007	Thigh and calf InterMAT, cm^3^	06//12	20.9 (2.8)	23.8 (4.2)	−0.84	Unilateral limb suspension	28	MRI	–	Thigh: 166 (50) Midcalf: 30 (6.9)	Thigh: 190 (60) Midcalf: 36 (8.6)	
Ogawa, Belavý et al., 2020	Thigh IntraMAT and InterMAT, cm^3^	8/0	34.0 (7.1)	24.6 (2.5)	−1.1	Bed rest	56	MRI	Yes	**IntraMAT** Quadriceps femoris: 387.5 (51.2) Vastus lateralis: 133.4 (16.3) Vastus medialis: 103.7 (23.0) Vastus intermedius: 109.6 (27.7) Hamstrings: 183.0 (38.0) Whole thigh: 834.6 (67.3) Rectus femoris: 40.8 (7.9) Biceps femoris short head: 27.6 (8.3) Biceps femoris long head: 51.9 (12.3) Semitendinosus: 48.3 (16.0) Semimembranosus: 55.2 (12.7) Adductor longus: 29.7 (5.1) Adductor magnus: 126.9 (16.5) Adductor brevis: 28.7 (6.9) Sartorius: 53.9 (17.4) Gracilis: 24.9 (5.1) Hip adductors: 264.1 (32.3) **InterMAT** Thigh: 375.0 (128.4)	**IntraMAT** Quadriceps femoris: 344.0 (71.8) Vastus lateralis: 107.0 (16.8) Vastus medialis: 96.6 (26.4) Vastus intermedius: 109.0 (29.2) Hamstrings: 156.9 (44.2) Whole thigh: 721.7 (161.7) Rectus femoris: 38.0 (9.2) Biceps femoris short head: 24.1 (8.9) Biceps femoris long head: 41.6 (15.4) Semitendinosus: 36.4 (9.6) Semimembranosus: 54.7 (15.8) Adductor longus: 27.2 (6.8) Adductor magnus: 101.0 (23.5) Adductor brevis: 28.6 (18.5) Sartorius: 47.9 (19.1) Gracilis: 20.8 (4.1) Hip adductors: 215.3 (49.8) **InterMAT** Thigh: 355.5 (182.4)	**IntraMAT** Quadriceps femoris: −48.2 (*P* < 0.05) Vastus lateralis: −26.4 (*P* < 0.05) Vastus medialis: −6.0 (*P* > 0.05) Vastus intermedius: −13.0 (*P* < 0.05) Hamstrings: −26.2 (*P* < 0.05) Whole thigh: −111.2 (*P* < 0.05) Rectus femoris: −2.8 (*P* < 0.05) Biceps femoris short head: −3.5 (*P* < 0.05) Biceps femoris long head: −10.3 (*P* < 0.05) Semitendinosus: −11.8 (*P* > 0.05) Semimembranosus: −0.5 (*P* < 0.05) Adductor longus: −2.7 (*P* < 0.05) Adductor magnus: −22.3 (*P* < 0.05) Adductor brevis: +0.4 (*P* > 0.05) Sartorius: −6.0 (*P* < 0.05) Gracilis: −4.1 (*P* < 0.05) Hip adductors: −41.4 (*P* < 0.05) **InterMAT** Thigh: −15.3 (*P* > 0.05)
Rudwill et al., 2018	Calf IntraMAT, %	9/0	31.0 (6.3)	23.8 (1.5)	−0.9	Bed rest	21	MRI	Yes	4.5 (0.3)	4.8 (0.6)	*P* < 0.04
Wesselink et al., 2025	Lumbar IntraMAT, %	16//8	–	–	–	Bed rest	59	MRI	–			Lumbar multifundus: +18.7 (15.7) Lumbar erector spinae: +5.4 (5.9) Quadratus lumborum: +4.5 (4.1); *P* < 0.001

*Note*: Data are expressed as the mean (SD). Abbreviations: BMI, body mass index; IntraMAT, intramuscular; InterMAT, intermuscular.

### 3.1 Effects of bed rest on IntraMAT

Bed rest protocols consistently demonstrated site‐specific increases in IntraMAT, particularly in the lumbar region and lower extremities. In a 60 day head‐down bed rest study in healthy young men, calf IntraMAT increased non‐significantly by 2.7% in the gastrosoleus complex (*P* = 0.54) despite a mean body weight loss of 3.3 kg (Bergouignan et al., 2009). Likewise, Rudwill et al. (2018) observed a modest increase in calf IntraMAT from 4.5% ± 0.1% to 4.8% ± 0.2% (*P* = 0.04) after 21 days of bed rest, also accompanied by a small mean weight loss (−0.9 kg; Rudwill et al., 2018). In the anterior thigh, Fuchs et al. (2024) reported an increase in IntraMAT levels after 14 days of bed rest, from baseline (4.4% ± 1.2%) to post‐intervention (4.5% ± 1.4%; *P* = 0.02; Fuchs et al., 2024). In a 56 day bed rest study, (Ogawa, Belavý et al., 2020) evaluated changes in IntraMAT and InterMAT volume across multiple individual thigh muscles (Ogawa, Belavý et al., 2020). Although none of the changes reported reached statistical significance (all *P* > 0.05), consistent trends towards reduction were observed. In particular, the rectus femoris InterMAT volume decreased from 40.8 ± 7.9 to 38.0 ± 9.2 cm^3^ (∆ = −2.8 cm^3^), the vastus lateralis IntraMAT volume from 133.4 ± 16.3 to 107.0 ± 16.8 cm^3^ (∆ = −26.4 cm^3^) and the vastus intermedius from 109.6 ± 27.7 to 109.0 ± 29.2 cm^3^ (∆ = −13.0 cm^3^). Likewise, the vastus medialis declined from 103.7 ± 23.0 to 96.6 ± 26.4 cm^3^ (∆ = −6.0 cm^3^), while the overall quadriceps femoris reduced from 387.5 ± 51.2 to 344.0 ± 71.8 cm^3^ (∆ = −48.2 cm^3^), with an associated increase in IntraMAT content from 15.6% ± 2.2% to 16.0% ± 3.2% (∆ = +0.4%; *P* < 0.05). In the posterior compartment, the biceps femoris short head decreased from 27.6 ± 8.3 to 24.1 ± 8.9 cm^3^ (∆ = −3.5 cm^3^) and the biceps femoris long head from 51.9 ± 12.3 to 41.6 ± 15.4 cm^3^ (∆ = −10.3 cm^3^; *P* < 0.05). The semitendinosus dropped insignificantly from 48.3 ± 16.0 to 36.4 ± 9.6 cm^3^ (∆ = −11.8 cm^3^; *P* > 0.05), while the semimembranosus showed a statistically significant change (from 55.2 ± 12.7 to 54.7 ± 15.8 cm^3^; ∆ = −0.5 cm^3^; *P* < 0.05). The total hamstrings IntraMAT volume decreased from 183.0 ± 38.0 to 156.9 ± 44.2 cm^3^ (∆ = −26.2 cm^3^), with a slight but significant reduction in IntraMAT content from 18.3% ± 3.3% to 17.5% ± 4.4% (∆ = −0.8%; *P* < 0.05). The adductor muscle group also showed declines: the adductor longus from 29.7 ± 5.1 to 27.2 ± 6.8 cm^3^ (∆ = −2.7 cm^3^), adductor magnus from 126.9 ± 16.5 to 101.0 ± 23.5 cm^3^ (∆ = −22.3 cm^3^) and adductor brevis from 28.7 ± 6.9 to 26.6 ± 18.5 cm^3^ (∆ = −2.1 cm^3^; *P* < 0.05). The total hip adductors IntraMAT volume decreased from 264.1 ± 32.3 to 215.3 ± 49.8 cm^3^ (∆ = −41.4 cm^3^), with IntraMAT content declining from 19.2% ± 1.4% to 17.4% ± 3.4% (∆ = −1.8%; *P* < 0.05). Other muscles also exhibited significant reductions: the sartorius from 53.9 ± 17.4 to 47.9 ± 19.1 cm^3^ (∆ = −6.0 cm^3^) and the gracilis from 24.9 ± 5.1 to 20.8 ± 4.1 cm^3^ (∆ = −4.1 cm^3^; *P* < 0.05). Finally, the whole thigh IntraMAT volume declined from 834.6 ± 67.3 to 721.7 ± 161.7 cm^3^ (∆ = −111.2 cm^3^), accompanied by a small but statistically insignificant decrease in IntraMAT content from 17.3% ± 1.1% to 17.0% ± 3.7% (∆ = −0.3%; *P* > 0.05). Wesselink et al. (2025) reported a mean increase of 18.7% ± 15.7% IntraMAT in the lumbar multifidus, 5.4% ± 5.9% in the erector spinae and 4.5% ± 4.1% (all *P* < 0.001) in the quadratus lumborum after 59 days of bed rest (Wesselink et al., 2025).

### 3.2 Effects of immobilization on IntraMAT

Protocols using unilateral lower‐limb immobilization revealed heterogeneous changes in IntraMAT. Gerlach et al. (2017) observed the effect of unilateral lower‐limb immobilization on calf fat fraction changes after 60 days. In the medial gastrocnemius, IntraMAT decreased slightly, from 10.2% ± 1.2% to 9.9% ± 1.2% (∆ = −0.3%), without reaching statistical significance (*P* > 0.05). The lateral gastrocnemius showed a significant reduction, from 10.8% ± 1.4% to 10.3% ± 1.0% (∆ = −0.5%; *P* < 0.05). Additionally, the soleus muscle exhibited a non‐significant increase in IntraMAT, from 10.9% ± 1.5% to 11.2% ± 2.0% (∆ = +0.3%; *P* > 0.05). Regarding muscle groups, the ventral muscle group IntraMAT decreased from 10.8% ± 0.8% to 10.4% ± 0.9% (∆ = −0.4%; *P* < 0.05) and the central muscle group showed a similar trend, decreasing from 10.9% ± 0.9% to 10.3% ± 0.9% (∆ = −0.6%; *P* < 0.05; Gerlach et al., 2017). Holloway et al. (2019) reported a marked increase in thigh IntraMAT (by 12.8% ± 6.1%; *P* < 0.05) after only 28 days of unilateral lower‐limb immobilization (Holloway et al., 2019).

### 3.3 Effects of bed rest on InterMAT

Evidence for InterMAT accumulation following bed rest is limited and shows mixed results depending on muscle region. In the thigh, Ogawa, Belavý et al. (2020) reported a reduction of 15.3 cm^3^ (from 375.0 ± 128.4 to 355.5 ± 182.4 cm^3^) in total InterMAT volume after 56 days of bed rest, albeit not reaching statistical significance (*P* > 0.05). In contrast, De Martino et al. (2021) found increases in lumbar InterMAT with 59 days of bed rest, particularly in multifidus and erector spinae (De Martino et al., 2021). In the lumbar multifidus muscle, values increased consistently across all intervertebral disc levels. At the L1–L2 level, the mean lumbar multifidus InterMAT content rose from 14.0% ± 7.4% to 15.2% ± 8.8% (∆ = +1.2%; *P* = 0.005), at L2–L3 it increased from 12.6% ± 6.9% to 13.8% ± 7.1% (∆ = +1.2%; *P* < 0.001) and at L3–L4 from 15.2% ± 6.5% to 17.4% ± 6.8% (∆ = +2.2%; *P* < 0.001). At the L4–L5 level, the lumbar multifidus IntraMAT percentage increased from 20.7% ± 10.6% to 23.5% ± 10.9% (∆ = +2.8%; *P* < 0.001) and at L5–S1 from 24.1% ± 8.3% to 29.4 ± 9.2% (∆ = +5.3%; *P* < 0.001). Likewise, the lumbar erector spinae also showed significant increases in InterMAT across all disc levels. At the L1–L2 intervertebral disc, InterMAT increased from 9.4% ± 3.4% to 11.1% ± 4.3% (∆ = +1.7%; *P* < 0.001), at L2–L3 from 11.3% ± 4.8% to 13.7% ± 6.2% (∆ = +2.4%; *P* < 0.001) and at L3–L4, from 12.7% ± 6.6% to 15.5% ± 8.0% (∆ = +2.8%; *P* < 0.001). A further increase was observed at L4–L5, with values increasing from 21.2% ± 12.6% to 24.3% ± 12.8% (∆ = +3.1%; *P* < 0.001), and at L5/S1 from 37.8% ± 13.3% to 41.1% ± 15.1% (∆ = +3.3%; *P* < 0.001).

### 3.4 Effects of immobilization on InterMAT

Manini et al. (2007) investigated changes in thigh and calf InterMAT following 28 days of unilateral lower‐limb immobilization in young adults (Manini et al., 2007). Their results demonstrated a significant increase in thigh InterMAT, rising from 166 ± 50 to 190 ± 60 cm^3^ (∆ = +24 cm^3^; *P* = 0.005), and in mid‐calf InterMAT, which increased from 30 ± 6.9 to 36 ± 8.6 cm^3^ (∆ = +6 cm^3^; *P* = 0.001). The relative increase in InterMAT was greater in the calf (+20%) than in the thigh (+14.5%; *P* = 0.005).

### 3.5 Risk‐of‐bias assessment

**FIGURE 2:**
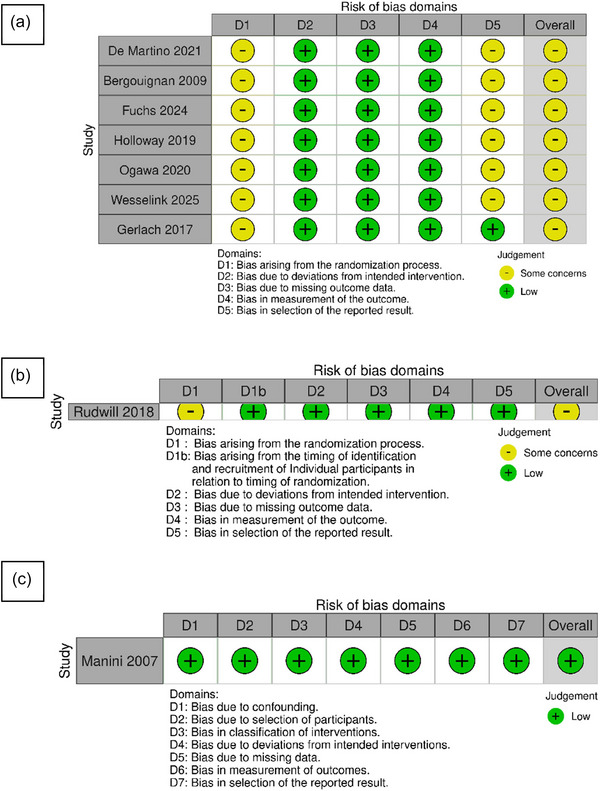
Risk‐of‐bias assessment of the included studies.

Assessment of the risk of bias for each study is presented in Figure 2. Using the RoB2 tool, all included studies had some concerns (Figure 2a). Likewise, ROBINS‐I was used for one study that displayed some concerns (Figure 2b), and a crossover randomized controlled trial had a low overall risk (Figure 2c). Primarily, these concerns were related to the lack of randomization process and blinding.

## 4 DISCUSSION

This systematic review suggests that muscle disuse can promote increases in IntraMAT and InterMAT, although their magnitude, distribution, and temporal dynamics depend on the experimental model and muscle tissue examined via MRI.

### 4.1 Impact of bed rest

During bed rest, consistent increases in IntraMAT and InterMAT in paraspinal muscles highlight the susceptibility of these regions to unloading. These muscles are enriched in type I fibres, which display metabolic regulation that favours lipid deposition in conditions of reduced activity (Johnson et al., 1973; Komiya et al., 2017; Regev et al., 2010). The soleus, chronically engaged during stance and locomotion (Neptune et al., 2001), undergoes profound transcriptomic and metabolic remodelling when deprived of tonic activation, including reductions in oxidative enzyme activity and mitochondrial function that could impair fatty acid oxidation (Fajardo et al., 2017). Such metabolic shifts provide a mechanistic basis for intramyocellular lipid accumulation observed in bed rest trials (Komiya et al., 2017). Likewise, paraspinal muscles (essential for postural control) demonstrate marked sensitivity to inactivity, making them particularly prone to fat accumulation (Teichtahl et al., 2015). Thus, bed rest‐induced fat infiltration in paraspinal and soleus muscles may compromise postural stability and metabolic health.

In contrast, (Ogawa, Belavý et al., 2020) reported a trend towards reductions in thigh IntraMAT and InterMAT. This discrepancy could be explained by several factors, including a lower oxidative muscle fibre content of quadriceps and hamstrings, redistribution of fat towards visceral depots during inactivity (Belavy et al., 2014; Jacob et al., 2015; Moreno‐Justicia et al., 2025), and dietary control; the trial enforced a strictly standardized diet that maintained energy balance. These differences are crucial, considering that positive energy balance may drive IntraMAT and InterMAT accumulation independent of unloading. However, it is noteworthy that Rudwill et al. (2018) and Bergouignan et al. (2009) observed increases in IntraMAT despite mean weight losses of 0.9 and 3.3 kg, respectively. Interestingly, in the study by Bergouignan et al. (2009), calorie intake was significantly reduced after 60 days of bed rest, yet gastrosoleus IntraMAT increased (non‐significantly). Importantly, the bed rest group showed a small negative energy balance (∼0.54 ± 0.38 MJ/day), corresponding to ∼88.5% agreement between estimated and measured energy balance. In addition, Rudwill et al. (2018) reported a significant rise in calf IntraMAT after 21 days of bed rest while participants were maintained in near‐energy balance via individualized diets set at 1.2 × resting metabolic rate, illustrating that IntraMAT accrual may occur despite modest lean soft tissue losses and without evidence of meaningful energy deficit. These findings suggest that: (1) IntraMAT accumulation may occur even during negative energy balance and body weight loss; and (2) caloric reductions during bed rest can exceed energy expenditure declines, resulting in mild energy deficit, reinforcing point 1. Nevertheless, using a standardized dietary control in future studies to isolate mechanisms could be a strong tool for causal inferences, and mechanistic work to disentangle how IntraMAT can accumulate in the presence of body weight losses may be warranted.

Methodological variability also warrants consideration. For instance, conventional T1‐weighted MRI with manual segmentation is less sensitive to subtle intramyocellular lipid shifts and could overestimate IntraMAT, whereas Dixon‐based fat‐fraction imaging provides greater precision and might consistently demonstrate adipose accumulation (Bolsterlee et al., 2021; Ogawa, Yoshiko et al., 2020; Wokke et al., 2013). Thus, despite variable findings, mechanistic evidence indicates that unloading could activate fibro‐adipogenic progenitors and adipocyte‐related proteins (e.g. perilipin, FABP4), while suppressing oxidative capacity and lipid utilization. This combination could create a permissive environment for ectopic muscle fat deposition, a process consistently observed across human and animal models (Gaster, 2011; Pagano et al., 2018; Tan et al., 2025). Collectively, these mechanisms could explain why paraspinal and soleus muscles consistently accumulated IntraMAT and InterMAT during bed rest, whereas thigh responses remained more variable and context dependent. Methodological heterogeneity highlights the need for advanced imaging to quantify disuse‐induced fat infiltration accurately, informing targeted interventions.

### 4.2 Impact of unilateral immobilization

Unilateral lower‐limb immobilization has been studied using diverse models, each imposing distinct mechanical constraints. Knee bracing, which restricts joint motion and predominantly inactivates the quadriceps, could often lead to rapid IntraMAT accumulation in thigh muscles (Holloway et al., 2019). In contrast, the Hephaistos orthosis, which unloads the knee and ankle while still permitting partial weight‐bearing, allows intermittent gastrocnemius activation; this residual recruitment may facilitate lipid utilization and help to explain reductions in gastrocnemius IntraMAT despite concurrent increases in the soleus (Gerlach et al., 2017). This is crucial to consider, given that model‐specific constraints, such as knee bracing, could partly preserve muscle activity in unloaded areas (e.g. gastrocnemius), leading to variable fat accumulation in comparison to complete unloading in suspension models, which may induce complete unloading and drive InterMAT increases in both thigh and calf compartments (Manini et al., 2007). Thus, immobilization model‐specific constraints may lead to variable fat accumulation, where complete unloading could exacerbate InterMAT deposition.

The heterogeneity of these outcomes reflects not only differences in model design and unloading intensity, but also intrinsic skeletal muscle biology. The soleus, a slow‐twitch muscle rich in oxidative type I fibres, could be particularly lipid‐prone during muscle disuse, whereas the gastrocnemius, a fast‐twitch muscle dominated by glycolytic type II muscle fibres, often shows slower or inconsistent changes (Soendenbroe et al., 2025; Vasileiadou et al., 2023). Moreover, prolonged immobilization can promote fibre‐type transitions from oxidative type I muscle fibres towards more glycolytic type II fibre phenotypes, further reducing oxidative capacity and exacerbating lipid accumulation in slow‐twitch compartments (Stein & Wade, 2005). Taken together, these findings indicate that the focal nature of unilateral lower‐limb immobilization, combined with partial preservation of activity in some protocols, may result in more variable remodelling of IntraMAT and InterMAT compared with the more uniform vulnerability observed in systemic bed rest.

### 4.3 Future directions

Future research should focus on longitudinal studies, which are particularly needed to capture both early and late phases of remodelling, ideally incorporating molecular markers, such as fibro‐adipogenic progenitor activity, mitochondrial dynamics, and lipid droplet turnover. Given the influence of energy balance on ectopic muscle fat deposition, future protocols should also rigorously control and report dietary intake, because differences in caloric supply could confound the interpretation of muscle disuse‐specific effects. Added to this, considering some evidence around IntraMAT accumulation despite caloric reductions, mechanistic research should be incorporated to investigate this phenomenon. Moreover, model‐specific differences, such as systemic unloading in bed rest vs. immobilization, may yield variable thigh responses, warranting work pertaining to within‐study explorations between these disuse models. Finally, clinical trials should link imaging‐derived measures of IntraMAT and InterMAT with functional outcomes to establish their prognostic and therapeutic relevance. In parallel, targeted interventions, such as exercise, warrant further evaluation, because they have already demonstrated efficacy in attenuating these changes during short‐term bed rest (Mastrandrea et al., 2025; Prokopidis et al., 2025).

### 4.4 Strengths and limitations

This systematic review integrates findings from two major disuse models and distinguishes between IntraMAT and InterMAT compartments, providing a comprehensive narrative synthesis of available evidence. However, several limitations should be acknowledged: (1) relatively few immobilization studies exist; (2) most included small, homogeneous samples of young men; (3) imaging modalities and analytical approaches varied substantially; (4) several trials did not control rigorously for dietary intake, which might confound observed changes in muscle fat infiltration; (5) follow‐up durations were often short, potentially underestimating cumulative remodelling; (6) few studies directly assessed functional correlations, limiting translational interpretation; (7) differences in the duration of disuse protocols complicate synthesis across studies; and (8) heterogeneity of imaging modalities. In particular, heterogeneity in MRI techniques, including pulse sequences (e.g. T1‐weighted vs. Dixon), field strengths and fat quantification methods, coimpact the sensitivity and comparability of IntraMAT and InterMAT measurements (Huijgen et al., 2019; Wokke et al., 2013). Such variability might complicate meta‐analyses and narrative syntheses and the interpretation of site‐specific fat infiltration, particularly in lumbar and thigh muscles. These limitations restrict generalizability, particularly to older adults, women and patients with chronic disease; populations for whom the clinical implications are most relevant.

## 5 CONCLUSION

Muscle disuse is consistently associated with increases in IntraMAT and InterMAT, but the magnitude and distribution of these changes are highly dependent on the model and the muscle tissue. Bed rest preferentially promotes accumulation in paraspinal and distal muscles, whereas thigh responses remain inconsistent. Unilateral immobilization produces more heterogeneous outcomes, with divergent responses even within the calf, highlighting the role of fibre‐type composition and unloading characteristics. Preservation of muscle structure should be an explicit target in preventive and rehabilitative strategies, alongside maintenance of muscle strength and function. Future research should prioritize standardized imaging protocols, rigorous control of dietary intake, mechanistic insights, and longitudinal designs incorporating both molecular markers and functional outcomes, particularly in older adults and clinical populations that may be more vulnerable to muscle disuse‐induced impairments.

## AUTHOR CONTRIBUTIONS

Konstantinos Prokopidis conceptualized the idea of this project. Konstantinos Prokopidis and Julia Margarita Reyes performed the screening and extracted the data from the included studies. Risk‐of‐bias assessment was performed by Konstantinos Prokopidis and Julia Margarita Reyes. Konstantinos Prokopidis and Julia Margarita Reyes wrote the manuscript. Gustavo Duque revised the manuscript. All authors approved the final version of the manuscript and agree to be accountable for all aspects of the work in ensuring that questions related to the accuracy or integrity of any part of the work are appropriately investigated and resolved. All persons designated as authors qualify for authorship, and all those who qualify for authorship are listed.

## CONFLICT OF INTEREST

None declared.

## FUNDING INFORMATION

None.

## Data Availability

Data are available upon request.

## References

[eph70180-bib-0001] Addison, O. , Marcus, R. L. , LaStayo, P. C. , & Ryan, A. S. (2014). Intermuscular fat: A review of the consequences and causes. International Journal of Endocrinology, 2014, 1–11. 10.1155/2014/309570 PMC3910392 24527032

[eph70180-bib-0002] Belavy, D. L. , Mohlig, M. , Pfeiffer, A. F. , Felsenberg, D. , & Armbrecht, G. (2014). Preferential deposition of visceral adipose tissue occurs due to physical inactivity. International Journal of Obesity (2005), 38(11), 1478–1480. 24522244 10.1038/ijo.2014.26

[eph70180-bib-0003] Bergouignan, A. , Trudel, G. , Simon, C. , Chopard, A. , Schoeller, D. A. , Momken, I. , Votruba, S. B. , Desage, M. , Burdge, G. C. , & Gauquelin‐Koch, G. (2009). Physical inactivity differentially alters dietary oleate and palmitate trafficking. Diabetes, 58(2), 367–376. 19017764 10.2337/db08-0263 PMC2628610

[eph70180-bib-0004] Bolsterlee, B. , Bye, E. A. , Eguchi, J. , Thom, J. & , & Herbert, R. D. (2021). MRI‐based measurement of effects of strength training on intramuscular fat in people with and without spinal cord injury. Medicine and Science in Sports and Exercise, 53(6), 1270–1275. 33986231 10.1249/MSS.0000000000002568

[eph70180-bib-0005] Brooks, N. , Cloutier, G. J. , Cadena, S. M. , Layne, J. E. , Nelsen, C. A. , Freed, A. M. , Roubenoff, R. , & Castaneda‐Sceppa, C. (2008). Resistance training and timed essential amino acids protect against the loss of muscle mass and strength during 28 days of bed rest and energy deficit. Journal of Applied Physiology, 105(1), 241–248. 18483167 10.1152/japplphysiol.01346.2007 PMC2494840

[eph70180-bib-0006] Carter, C. S. , Justice, J. N. , & Thompson, L. (2019). Lipotoxicity, aging, and muscle contractility: Does fiber type matter? Geroscience, 41(3), 297–308. 31227962 10.1007/s11357-019-00077-z PMC6702511

[eph70180-bib-0007] Cree, M. G. , Paddon‐Jones, D. , Bailey, M. , & Newcomer, B. R. (2005). 28 Day bed‐rest with hypercortisolemia induces peripheral insulin resistance and increases intramuscular and liver fat. Diabetes, 54, A378. 10.1016/j.metabol.2009.09.014 PMC2856785 19919871

[eph70180-bib-0008] Deane, C. S. , Piasecki, M. , & Atherton, P. J. (2024). Skeletal muscle immobilisation‐induced atrophy: Mechanistic insights from human studies. Clinical Science, 138(12), 741–756. 38895777 10.1042/CS20231198 PMC11186857

[eph70180-bib-0009] Demangel, R. , Treffel, L. , Py, G. , Brioche, T. , Pagano, A. F. , Bareille, M. P. , Beck, A. , Pessemesse, L. , Candau, R. , & Gharib, C. (2017). Early structural and functional signature of 3‐day human skeletal muscle disuse using the dry immersion model. The Journal of Physiology, 595(13), 4301–4315. 28326563 10.1113/JP273895 PMC5491890

[eph70180-bib-0010] De Martino, E. , Hides, J. , Elliott, J. M. , Hoggarth, M. , Zange, J. , Lindsay, K. , Debuse, D. , Winnard, A. , Beard, D. , & Cook, J. A. (2021). Lumbar muscle atrophy and increased relative intramuscular lipid concentration are not mitigated by daily artificial gravity after 60‐day head‐down tilt bed rest. Journal of Applied Physiology, 131(1), 356–368. 34080918 10.1152/japplphysiol.00990.2020

[eph70180-bib-0011] De Martino, E. , Hides, J. , Elliott, J. M. , Hoggarth, M. A. , Zange, J. , Lindsay, K. , Debuse, D. , Winnard, A. , Beard, D. , & Cook, J. A. (2022a). The effects of reconditioning exercises following prolonged bed rest on lumbopelvic muscle volume and accumulation of paraspinal muscle fat. Frontiers in Physiology, 13, 862793. 35774286 10.3389/fphys.2022.862793 PMC9237402

[eph70180-bib-0012] De Martino, E. , Hides, J. , Elliott, J. M. , Hoggarth, M. A. , Zange, J. , Lindsay, K. , Debuse, D. , Winnard, A. , Beard, D. , & Cook, J. A. (2022b). Intramuscular lipid concentration increased in localized regions of the lumbar muscles following 60 day bedrest. The Spine Journal, 22(4), 616–628. 34813960 10.1016/j.spinee.2021.11.007

[eph70180-bib-0013] Di Girolamo, F. G. , Fiotti, N. , Milanović, Z. , Situlin, R. , Mearelli, F. , Vinci, P. , Šimunič, B. , Pišot, R. , Narici, M. , & Biolo, G. (2021). The aging muscle in experimental bed rest: A systematic review and meta‐analysis. Frontiers in Nutrition, 8, 633987. 34422875 10.3389/fnut.2021.633987 PMC8371327

[eph70180-bib-0014] Dirks, M. L. , Wall, B. T. , Van De Valk, B. , Holloway, T. M. , Holloway, G. P. , Chabowski, A. , Goossens, G. H. , & van Loon, L. J. (2016). One week of bed rest leads to substantial muscle atrophy and induces whole‐body insulin resistance in the absence of skeletal muscle lipid accumulation. Diabetes, 65(10), 2862–2875. 27358494 10.2337/db15-1661

[eph70180-bib-0015] Eggelbusch, M. , Charlton, B. T. , Bosutti, A. , Ganse, B. , Giakoumaki, I. , Grootemaat, A. E. , Hendrickse, P. W. , Jaspers, Y. , Kemp, S. , & Kerkhoff, T. J. (2024). The impact of bed rest on human skeletal muscle metabolism. Cell Reports Medicine, 5(1), 101372. 38232697 10.1016/j.xcrm.2023.101372 PMC10829795

[eph70180-bib-0016] Erley, J. , Roedl, K. , Ozga, A.‐K. , de Heer, G. , Schubert, N. , Breckow, J. , Burdelski, C. , Tahir, E. , Kluge, S. , & Huber, T. B. (2024). Dual‐Energy CT muscle fat fraction as a new imaging biomarker of body composition and survival predictor in critically ill patients. European Radiology, 34(11), 7408–7418. 38777903 10.1007/s00330-024-10779-4 PMC11519288

[eph70180-bib-0017] Fajardo, V. A. , Mikhaeil, J. S. , Leveille, C. F. , Saint, C. , & LeBlanc, P. J. (2017). Cardiolipin content, linoleic acid composition, and tafazzin expression in response to skeletal muscle overload and unload stimuli. Scientific Reports, 7(1), 2060. 28515468 10.1038/s41598-017-02089-1 PMC5435726

[eph70180-bib-0018] Fuchs, C. J. , Hermans, W. J. , Nyakayiru, J. , Weijzen, M. E. , Smeets, J. S. , Aussieker, T. , Senden, J. M. , Wodzig, W. K. , Snijders, T. , & Verdijk, L. B. (2024). Daily blood flow restriction does not preserve muscle mass and strength during 2 weeks of bed rest. The Journal of Physiology, 603(13), 3837–3856. 38411283 10.1113/JP286065 PMC12306398

[eph70180-bib-0019] Gaster, M. (2011). Mitochondrial mass is inversely correlated to complete lipid oxidation in human myotubes. Biochemical and Biophysical Research Communications, 404(4), 1023–1028. 21187069 10.1016/j.bbrc.2010.12.102

[eph70180-bib-0020] Gerlach, D. , Schopen, K. , Linz, P. , Johannes, B. , Titze, J. , Zange, J. , & Rittweger, J. (2017). Atrophy of calf muscles by unloading results in an increase of tissue sodium concentration and fat fraction decrease: A ^23^Na MRI physiology study. European Journal of Applied Physiology, 117(8), 1585–1595. 28534200 10.1007/s00421-017-3647-4

[eph70180-bib-0021] Higgins, J. P. , Altman, D. G. , Gøtzsche, P. C. , Jüni, P. , Moher, D. , Oxman, A. D. , Savović, J. , Schulz, K. F. , Weeks, L. , & Sterne, J. A. (2011). The Cochrane Collaboration's tool for assessing risk of bias in randomised trials. British Medical Journal (Clinical research ed.), 343(oct18 2), d5928–d5928. 10.1136/bmj.d5928 PMC3196245 22008217

[eph70180-bib-0022] Holloway, T. M. , McGlory, C. , McKellar, S. , Morgan, A. , Hamill, M. , Afeyan, R. , Comb, W. , Confer, S. , Zhao, P. , & Hinton, M. (2019). A novel amino acid composition ameliorates short‐term muscle disuse atrophy in healthy young men. Frontiers in Nutrition, 6, 105. 31355205 10.3389/fnut.2019.00105 PMC6636393

[eph70180-bib-0023] Huijgen, W. H. , van Rijswijk, C. S. , & Bloem, J. L. (2019). Is fat suppression in T1 and T2 FSE with mDixon superior to the frequency selection‐based SPAIR technique in musculoskeletal tumor imaging? Skeletal Radiology, 48(12), 1905–1914. 31154494 10.1007/s00256-019-03227-8 PMC6813285

[eph70180-bib-0024] Jacob, M. A. , Matt, S. S. , Elias, C. C. , Kendra, O. D. , Alexander, D. S. , & Brennan, T. J. (2015). Influence of hamstring fatigue on the estimated percentage of fast‐twitch muscle fibers for the vastus lateralis. Journal of Strength and Conditioning Research, 29, 3509–3516. 26219026 10.1519/JSC.0000000000000996

[eph70180-bib-0025] Johnson, M. A. , Polgar, J. , Weightman, D. , & Appleton, D. (1973). Data on the distribution of fibre types in thirty‐six human muscles: An autopsy study. Journal of the Neurological Sciences, 18(1), 111–129. 4120482 10.1016/0022-510x(73)90023-3

[eph70180-bib-0026] Komiya, Y. , Sawano, S. , Mashima, D. , Ichitsubo, R. , Nakamura, M. , Tatsumi, R. , Ikeuchi, Y. , & Mizunoya, W. (2017). Mouse soleus (slow) muscle shows greater intramyocellular lipid droplet accumulation than EDL (fast) muscle: Fiber type‐specific analysis. Journal of Muscle Research and Cell Motility, 38(2), 163–173. 28281032 10.1007/s10974-017-9468-6

[eph70180-bib-0027] Lees, M. J. , Prado, C. M. , Wischmeyer, P. E. , & Phillips, S. M. (2025). Skeletal muscle: A critical organ for survival and recovery in critical illness. Critical Care Clinics, 41(2), 299–312. 40021281 10.1016/j.ccc.2024.08.011

[eph70180-bib-0028] Manini, T. M. , Clark, B. C. , Nalls, M. A. , Goodpaster, B. H. , Ploutz‐Snyder, L. L. , & Harris, T. B. (2007). Reduced physical activity increases intermuscular adipose tissue in healthy young adults. The American Journal of Clinical Nutrition, 85(2), 377–384. 17284732 10.1093/ajcn/85.2.377

[eph70180-bib-0029] Marusic, U. , Narici, M. , Simunic, B. , Pisot, R. , & Ritzmann, R. (2021). Nonuniform loss of muscle strength and atrophy during bed rest: A systematic review. Journal of Applied Physiology, 131(1), 194–206. 33703945 10.1152/japplphysiol.00363.2020 PMC8325614

[eph70180-bib-0030] Mastrandrea, C. J. , Hajj‐Boutros, G. , Sonjak, V. , Hedge, E. T. , Gouspillou, G. , Hughson, R. L. , & Morais, J. A. (2025). Exercise attenuates bed rest‐induced increases in insulin resistance while alpha‐klotho increases in 55 to 65 year‐old women and men. Scientific Reports, 15(1), 26927. 40707556 10.1038/s41598-025-12770-5 PMC12290009

[eph70180-bib-0031] McNamara, K. P. , Greene, K. A. , Tooze, J. A. , Dang, J. , Khattab, K. , Lenchik, L. , & Weaver, A. A. (2019). Neck muscle changes following long‐duration spaceflight. Frontiers in Physiology, 10, 1115. 31572205 10.3389/fphys.2019.01115 PMC6753191

[eph70180-bib-0032] Moreno‐Justicia, R. , Van der Stede, T. , Stocks, B. , Laitila, J. , Seaborne, R. A. , Van de Loock, A. , Lievens, E. , Samodova, D. , Marin‐Arraiza, L. , Dmytriyeva, O. , Browaeys, R. , Van Vossel, K. , Moesgaard, L. , Yigit, N. , Anckaert, J. , Weyns, A. , Van Thienen, R. , Sahl, R. E. , Zanoteli, E. , … Deshmukh, A. S. (2025). Human skeletal muscle fiber heterogeneity beyond myosin heavy chains. Nature Communications, 16(1), 1764. 10.1038/s41467-025-56896-6 PMC11839989 39971958

[eph70180-bib-0033] Neptune, R. R. , Kautz, S. A. , & Zajac, F. E. (2001). Contributions of the individual ankle plantar flexors to support, forward progression and swing initiation during walking. Journal of Biomechanics, 34(11), 1387–1398. 11672713 10.1016/s0021-9290(01)00105-1

[eph70180-bib-0034] Ogawa, M. , Belavý, D. L. , Yoshiko, A. , Armbrecht, G. , Miokovic, T. , Felsenberg, D. , & Akima, H. (2020). Effects of 8 weeks of bed rest with or without resistance exercise intervention on the volume of the muscle tissue and the adipose tissues of the thigh. Physiological Reports, 8(18), e14560. 32951335 10.14814/phy2.14560 PMC7507449

[eph70180-bib-0035] Ogawa, M. , Yoshiko, A. , Tanaka, N. , Koike, T. , Oshida, Y. , & Akima, H. (2020). Comparing intramuscular adipose tissue on T1‐weighted and two‐point Dixon images. PLoS ONE, 15(4), e0231156. 32271803 10.1371/journal.pone.0231156 PMC7144956

[eph70180-bib-0036] Pagano, A. F. , Brioche, T. , Arc‐Chagnaud, C. , Demangel, R. , Chopard, A. , & Py, G. (2018). Short‐term disuse promotes fatty acid infiltration into skeletal muscle. Journal of Cachexia, Sarcopenia and Muscle, 9(2), 335–347. 29248005 10.1002/jcsm.12259 PMC5879967

[eph70180-bib-0037] Page, M. J. , McKenzie, J. E. , Bossuyt, P. M. , Boutron, I. , Hoffmann, T. C. , Mulrow, C. D. , Shamseer, L. , Tetzlaff, J. M. , Akl, E. A. , & Brennan, S. E. (2021). The PRISMA 2020 statement: An updated guideline for reporting systematic reviews. British Medical Journal (Clinical research ed.), 372, n71. 10.1136/bmj.n71 PMC8005924 33782057

[eph70180-bib-0038] Parry, S. M. , & Puthucheary, Z. A. (2015). The impact of extended bed rest on the musculoskeletal system in the critical care environment. Extreme Physiology & Medicine, 4(1), 16. 26457181 10.1186/s13728-015-0036-7 PMC4600281

[eph70180-bib-0039] Prokopidis, K. , Varanoske, A. N. , Veronese, N. , Kirk, B. , Triantafyllidis, K. K. , Giannaki, C. D. , Stavrinou, P. S. , Church, D. D. , & Duque, G. (2025). Effects of exercise with or without a hypocaloric diet on intermuscular and intramuscular fat: A systematic review. Aging Clinical and Experimental Research, 37(1), 183. 40490541 10.1007/s40520-025-03097-2 PMC12149019

[eph70180-bib-0040] Regev, G. J. , Kim, C. W. , Thacker, B. E. , Tomiya, A. , Garfin, S. R. , Ward, S. R. , & Lieber, R. L. (2010). Regional Myosin heavy chain distribution in selected paraspinal muscles. Spine, 35(13), 1265–1270. 20461040 10.1097/BRS.0b013e3181bfcd98 PMC2947743

[eph70180-bib-0041] Rommersbach, N. , Wirth, R. , Lueg, G. , Klimek, C. , Schnatmann, M. , Liermann, D. , Janssen, G. , Müller, M. J. , & Pourhassan, M. (2020). The impact of disease‐related immobilization on thigh muscle mass and strength in older hospitalized patients. BioMed Central Geriatrics, 20(1), 500. 33238889 10.1186/s12877-020-01873-5 PMC7687989

[eph70180-bib-0042] Rudwill, F. , O'gorman, D. , Lefai, E. , Chery, I. , Zahariev, A. , Normand, S. , Pagano, A. F. , Chopard, A. , Damiot, A. , & Laurens, C. (2018). Metabolic inflexibility is an early marker of bed‐rest–induced glucose intolerance even when fat mass is stable. The Journal of Clinical Endocrinology & Metabolism, 103(5), 1910–1920. 29546280 10.1210/jc.2017-02267 PMC7263792

[eph70180-bib-0043] Soendenbroe, C. , Svensson, R. B. , Mittendorfer, B. , Magnusson, S. P. , Mackey, A. L. , & Andersen, J. L. (2025). Morphological differences in myofibre size and shape: A comparative study of the soleus, gastrocnemius, triceps brachii and vastus lateralis in humans and mice. Journal of Anatomy, 248(1), 126–139. 40676763 10.1111/joa.70025 PMC12682596

[eph70180-bib-0044] Standley, R. A. , Distefano, G. , Pereira, S. L. , Tian, M. , Kelly, O. J. , Coen, P. M. , Deutz, N. E. , Wolfe, R. R. , & Goodpaster, B. H. (2017). Effects of β‐hydroxy‐β‐methylbutyrate on skeletal muscle mitochondrial content and dynamics, and lipids after 10 days of bed rest in older adults. Journal of Applied Physiology, 123(5), 1092–1100. 28705993 10.1152/japplphysiol.00192.2017

[eph70180-bib-0045] Stein, T. P. , & Wade, C. E. (2005). Metabolic consequences of muscle disuse atrophy. Journal of Nutrition, 135(7), 1824S–1828S. 15987873 10.1093/jn/135.7.1824S

[eph70180-bib-0046] Sterne, J. A. , Hernán, M. A. , Reeves, B. C. , Savović, J. , Berkman, N. D. , Viswanathan, M. , Henry, D. , Altman, D. G. , Ansari, M. T. , & Boutron, I. (2016). ROBINS‐I: A tool for assessing risk of bias in non‐randomised studies of interventions. British Medical Journal (Clinical research ed.), 355, i4919. 10.1136/bmj.i4919 PMC5062054 27733354

[eph70180-bib-0047] Tan, J. , Li, Y. , Zhang, J. , Qi, B. , Chen, J. & , & Sun, Y. (2025). Role of aberrant activated fibro/adipogenic progenitors and suppressed ferroptosis in disused skeletal muscle atrophy and fatty infiltration. Journal of molecular medicine (Berlin, Germany), 103(6), 713–724. 40316864 10.1007/s00109-025-02548-7

[eph70180-bib-0048] Teichtahl, A. J. , Urquhart, D. M. , Wang, Y. , Wluka, A. E. , Wijethilake, P. , O'Sullivan, R. , & Cicuttini, F. M. (2015). Fat infiltration of paraspinal muscles is associated with low back pain, disability, and structural abnormalities in community‐based adults. The Spine Journal: Official Journal of the North American Spine Society, 15(7), 1593–1601. 25828477 10.1016/j.spinee.2015.03.039

[eph70180-bib-0049] Tran, V. , De Martino, E. , Hides, J. , Cable, G. , Elliott, J. M. , Hoggarth, M. , Zange, J. , Lindsay, K. , Debuse, D. , & Winnard, A. (2021). Gluteal muscle atrophy and increased intramuscular lipid concentration are not mitigated by daily artificial gravity following 60‐day head‐down tilt bed rest. Frontiers in Physiology, 12, 745811. 34867450 10.3389/fphys.2021.745811 PMC8634875

[eph70180-bib-0050] Trudel, G. , Melkus, G. , Sheikh, A. , Ramsay, T. , & Laneuville, O. (2019). Marrow adipose tissue gradient is preserved through high protein diet and bed rest. A randomized crossover study. Bone Reports, 11, 100229. 31799339 10.1016/j.bonr.2019.100229 PMC6883331

[eph70180-bib-0051] Vasileiadou, O. , Nastos, G. G. , Chatzinikolaou, P. N. , Papoutsis, D. , Vrampa, D. I. , Methenitis, S. , & Margaritelis, N. V. (2023). Redox Profile of skeletal muscles: Implications for research design and interpretation. Antioxidants (Basel, Switzerland), 12(9), 1738. 37760040 10.3390/antiox12091738 PMC10525275

[eph70180-bib-0052] Wang, L. , Valencak, T. G. , & Shan, T. (2024). Fat infiltration in skeletal muscle: Influential triggers and regulatory mechanism. Iscience, 27(3), 109221. 38433917 10.1016/j.isci.2024.109221 PMC10907799

[eph70180-bib-0053] Wesselink, E. O. , Hides, J. , Elliott, J. M. , Hoggarth, M. , Weber, K. A. , Salomoni, S. E. , Tran, V. , Lindsay, K. , Hughes, L. , & Weber, T. (2025). New insights into the impact of bed rest on lumbopelvic muscles: A computer‐vision model approach to measure fat fraction changes. Journal of Applied Physiology, 138(1), 157–168. 39611883 10.1152/japplphysiol.00502.2024

[eph70180-bib-0054] Wokke, B. H. , Bos, C. , Reijnierse, M. , van Rijswijk, C. S. , Eggers, H. , Webb, A. , Verschuuren, J. J. , & Kan, H. E. (2013). Comparison of dixon and T1‐weighted MR methods to assess the degree of fat infiltration in duchenne muscular dystrophy patients. Journal of Magnetic Resonance Imaging, 38(3), 619–624. 23292884 10.1002/jmri.23998

[eph70180-bib-0055] Yoshiko, A. , Yamauchi, K. , Kato, T. , Ishida, K. , Koike, T. , Oshida, Y. , & Akima, H. (2018). Effects of post‐fracture non‐weight‐bearing immobilization on muscle atrophy, intramuscular and intermuscular adipose tissues in the thigh and calf. Skeletal Radiology, 47(11), 1541–1549. 29948037 10.1007/s00256-018-2985-6

[eph70180-bib-0056] Yuri, T. , Mura, N. , Hoshikawa, K. , Giambini, H. , Fujii, H. , & Kiyoshige, Y. (2021). Elastographic region of interest determination for muscle with fat infiltration. Clinical Interventions in Aging, 16, 645–653. 33907386 10.2147/CIA.S296981 PMC8064623

